# Artificial Intelligence in Head and Neck Cancer Diagnosis: A Comprehensive Review with Emphasis on Radiomics, Histopathological, and Molecular Applications

**DOI:** 10.3390/cancers16213623

**Published:** 2024-10-27

**Authors:** Giuseppe Broggi, Antonino Maniaci, Mario Lentini, Andrea Palicelli, Magda Zanelli, Maurizio Zizzo, Nektarios Koufopoulos, Serena Salzano, Manuel Mazzucchelli, Rosario Caltabiano

**Affiliations:** 1 Department of Medical and Surgical Sciences and Advanced Technologies “G.F. Ingrassia”, Anatomic Pathology, University of Catania, 95123 Catania, Italy; giuseppe.broggi@phd.unict.it (G.B.); sere.salzano@gmail.com (S.S.); manuel.mazzucchelli@virgilio.it (M.M.); rosario.caltabiano@unict.it (R.C.); 2 Department of Medicine and Surgery, University of Enna Kore, 94100 Enna, Italy; antonino.maniaci@unikore.it (A.M.); mario.lentini@unikore.it (M.L.); 3 ASP Ragusa-Hospital Giovanni Paolo II, 97100 Ragusa, Italy; 4 Pathology Unit, Azienda USL-IRCCS di Reggio Emilia, 42123 Reggio Emilia, Italy; andrea.palicelli@ausl.re.it; 5 Surgical Oncology Unit, Azienda USL-IRCCS di Reggio Emilia, 42123 Reggio Emilia, Italy; maurizio.zizzo@ausl.re.it; 6 Second Department of Pathology, Medical School, National and Kapodistrian University of Athens, Attikon University Hospital, 15772 Athens, Greece; nkoufo@med.uoa.gr

**Keywords:** head and neck cancer, artificial intelligence, machine learning, deep learning, diagnosis

## Abstract

The present review discusses the transformative role of AI in the diagnosis and management of head and neck cancers (HNCs). **Methods:** It explores how AI technologies, including ML, DL, and CNNs, are applied in various diagnostic tasks, such as medical imaging, molecular profiling, and predictive modeling. **Results:** This review highlights AI’s ability to improve diagnostic accuracy and efficiency, particularly in analyzing medical images like CT, MRI, and PET scans, where AI sometimes outperforms human radiologists. This paper also emphasizes AI’s application in histopathology, where algorithms assist in whole-slide image (WSI) analysis, tumor-infiltrating lymphocytes (TILs) quantification, and tumor segmentation. AI shows promise in identifying subtle or rare histopathological patterns and enhancing the precision of tumor grading and treatment planning. Furthermore, the integration of AI with molecular and genomic data aids in mutation analysis, prognosis, and personalized treatment strategies. **Conclusions:** Despite these advancements, the review identifies challenges in AI adoption, such as data standardization and model interpretability, and calls for further research to fully integrate AI into clinical practice for improved patient outcomes.

## Simple Summary

This article highlights how artificial intelligence (AI) is revolutionizing the diagnosis and treatment of head and neck cancers (HNCs). This article explores the use of various AI techniques, including machine learning (ML), deep learning (DL), and convolutional neural networks (CNNs), in medical imaging, molecular profiling, and predictive modeling. AI has demonstrated improved accuracy in analyzing CT, MRI, and PET scans, often surpassing human radiologists. It is also applied to histopathology, automating the analysis of whole-slide images and assisting in tumor grading and segmentation. Additionally, AI aids in molecular analysis, particularly in mutation detection and treatment personalization. While AI shows promising results in enhancing diagnostic precision and workflow efficiency, challenges such as data standardization and model transparency still remain.

## 1. Introduction

HNCs are among the most prevalent malignancies globally, representing approximately 4–5% of all cancers [1]. Early diagnosis and precise staging are essential for enhancing prognosis and lowering mortality rates associated with these tumors [1,2,3,4,5]. Traditionally, diagnosis relies on a combination of clinical examination, imaging techniques (such as CT, MRI, and PET scans) [2], histopathological analysis, and molecular testing [3]. Although effective, these methods are time-consuming and subject to variability based on the clinician’s expertise [4]. Recently, AI has emerged as a transformative tool in oncology, particularly in the diagnosis and management of head and neck cancers [6,7]. This review aims to summarize the latest advancements in AI applications for diagnosing head and neck tumors, focusing on studies published up to 2024.

AI in medical imaging and diagnosis includes various technologies such as ML, DL, CNNs, and NLP [7,8]. These technologies are designed to process vast amounts of data, identify patterns, and provide insights that can aid clinical decision-making [7]. In the realm of head and neck cancer, AI systems have been utilized for diverse diagnostic tasks, including image analysis, molecular profiling, and predictive modelling [9,10].

## 2. Materials and Methods

### 2.1. Search Strategy

The authors performed a thorough electronic literature search up to September 2024; the following databases were used to identify relevant articles: PubMed/MEDLINE, Cochrane Library (Cochrane Database of Systematic Reviews, Cochrane Central Register of Controlled Trials—CENTRAL), Web of Science (Science and Social Science Citation Index), and Scopus. The combination of non-MeSH/MeSH terms was as follows: (i) PubMed/MEDLINE: ((artificial intelligence [Title/abstract]) OR (machine learning [Title/abstract]) OR (Deep Learning [Title/abstract]) OR (Convolutional Neural Networks [Title/abstract]) OR (Radiomics [Title/abstract]) OR (Histopathology [Title/abstract]) OR (Molecular Diagnosis [Title/abstract]) OR (Genomics [Title/abstract]) AND (head and neck cancer [Title/abstract])). Filters applied: English; (ii) Cochrane Library: artificial intelligence in Title Abstract Keyword AND head and neck cancer in Title Abstract Keyword- (word variations have been searched). Language: English; (iii) Web of Science: artificial intelligence (Topic) AND head and neck cancer (Topic) OR machine learning (Topic) AND head and neck cancer (Topic) and English (Languages); and (iv) Scopus: (TITLE-ABS-KEY (artificial intelligence) AND TITLE-ABS-KEY (head and neck cancer) OR TITLE-ABS-KEY (deep learning) AND TITLE-ABS-KEY (head and neck cancer)) AND (LIMIT-TO (LANGUAGE, “English”)). Additionally, the reference lists of relevant studies were manually reviewed to identify any articles that may have been missed during the electronic search.

### 2.2. Inclusion and Exclusion Criteria

The timeframe for the selected studies ranged from January 2018 to September 2024. Articles not addressing head and neck tumors or those discussing them from a perspective other than the application of AI methods in radiomics and/or histopathological and molecular diagnosis and/or patient outcomes were excluded. Comments, opinions, perspectives, guidelines, editorials, case reports, previously published systematic reviews and/or meta-analyses, and papers in languages other than English were excluded. We also excluded publications available only as abstracts or those with text appearing too brief or non-informative. The inclusion criteria focused on articles in English with full texts available. We concentrated on articles that offered thorough summaries or in-depth discussions of AI applications in HNC diagnosis, outcome prediction, and clinical management. The inclusion criteria encompassed the following: (i) patients diagnosed with HNC; (ii) application of AI in radiomics and in the histopathological/molecular diagnosis of HNC; and (iii) application of AI in HNC patient outcomes.

## 3. Results

Our literature search identified 23 key studies (Table 1) that focus on the integration of AI in radiomics, diagnosis, and outcomes of HNC patients [11,12,13,14,15,16,17,18,19,20,21,22,23,24,25,26,27,28,29,30,31,32,33]. Briefly, AI-based radiomics is used to predict treatment responses and risks in neoplasms like NPC. Key limitations include small sample sizes, single-institution studies, and a lack of pathological confirmation. In the histopathological field, AI algorithms, particularly DL, are applied to detect and grade tumors and in distinguishing benign tumors from malignant ones. However, issues like limited datasets and unclear clinical applicability are common challenges. From a molecular point of view, AI is used to analyze gene expression and predict immune responses or HPV status in HNC. The main issues are the need for more comprehensive data and further verification of results. AI-driven analysis of ctDNA helps detect minimal residual disease and recurrence. These studies are often constrained by small patient numbers and retrospective data. AI models improve treatment outcome prediction, especially for chemotherapy response and toxicity. However, biases, limited data, and the need for more comprehensive studies remain major limitations.

In addition, pertinent data were extracted and arranged in a narrative manner.

## 4. AI in Medical Imaging for Head and Neck Cancer

One of the most notable applications of AI in diagnosing head and neck cancer is in the analysis of medical imaging. Various AI models, especially CNNs, have demonstrated remarkable accuracy in interpreting CT, MRI, and PET scans, sometimes outperforming human radiologists [34,35,36,37].

Several studies have underscored the effectiveness of AI in detecting and classifying tumors in the head and neck region. AI has also shown promise in analyzing PET scans, particularly in evaluating tumor metabolism and predicting treatment responses [34,35,36,37].

### 4.1. Texture Analysis and Radiomics 

**Figure 1:**
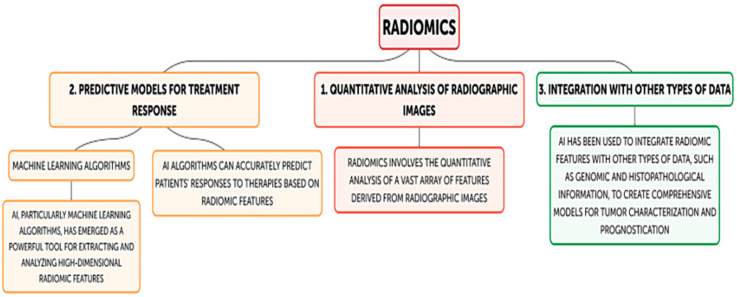
Flow-diagram of radiomics and AI application. Abbreviations: AI, artificial intelligence.

Analyzing radiomics properties and texture patterns taken from medical imaging data is one of the quickly developing uses of AI in head and neck cancer diagnoses. The quantitative study of a wide range of features obtained from radiographic images, including CT, MRI, and PET scans, is known as radiomics. Tumor phenotype, heterogeneity, and biological behavior can all be better understood by utilizing these features, which record information on tumor shape, intensity, texture, and spatial correlations (Figure 1).

However, conventional analytical techniques find it difficult to fully utilize the potential of radiomic data due to their enormous volume and complexity. AI has become a potent tool for extracting and analyzing these high-dimensional radiomic features, especially ML algorithms. This allows for more precise tumor characterization, treatment response prediction, and identification of subtle patterns that are invisible to the human eye. The creation of predictive models for treatment response in head and neck tumors is one of the main uses of AI in radiomics. AI algorithms may accurately predict patients’ responses to therapies like radiation, chemotherapy, and targeted drugs by analyzing radiomic characteristics from pre-treatment imaging data, as shown by several studies [38]. Some colleagues conducted a retrospective study in which they extracted radiomic signatures from various types of weighted images obtained through MRI of patients with NPC. They selected the most relevant radiomic features using the LASSO method (Least Absolute Shrinkage and Selection Operator) to create an effective model for predicting the early response of NPC patients to induction chemotherapy [11,12,13]. By identifying individuals who are more likely to benefit from intensive therapy, this technique may help clinicians avoid needless toxicities for patients who are unlikely to react. AI has been utilized to integrate radiomic characteristics with other forms of data, including genomic and histological information. Pre-treatment staging is a critical aspect of tumor diagnosis, significantly impacting prognosis. Traditional diagnostic approaches like biopsies, serological tests, and imaging provide tumor staging but are often localized, qualitative, and subjective. Radiomics offers a more objective, non-invasive alternative by analyzing imaging features to guide treatment selection and improve patient outcomes [39,40,41,42,43,44]. Studies have shown its potential to predict tumor stages and characteristics, such as in locally advanced laryngeal cancer or SCC, where radiomics-based models have demonstrated high accuracy and performance, enhancing preoperative staging and prognosis prediction.

Radiomics also helps predict tumor grade and lymph node status in oropharyngeal and oral cavity SCC. These techniques have proven effective in identifying histological grades and tumor differentiation, further improving the precision of cancer staging and treatment planning. Radiomics signatures can serve as a valuable complementary tool for preoperative staging. Some colleagues applied radiomics to predict tumor grade and lymph node status in oropharyngeal and oral cavity SCC [14].

### 4.2. AI for Early Detection and Screening

To improve prognosis and survival rates for head and neck malignancies, early identification is essential. More research is being carried out on AI as a useful tool for population-based screening programs and for identifying high-risk individuals for focused surveillance. Analyzing data from large-scale screening programs, such as imaging and genomic data, to find subtle patterns suggestive of precancerous lesions or early-stage malignancies is one promising use of AI. Some authors extracted 10,320 textural features by examining MRI images with multiple weightings from 242 patients with NPC who had received radiation therapy. They developed three prediction models using the RF method, all of which can dynamically forecast radiation-induced brain injury in advance [15]. This allows for early detection, enabling clinicians to implement preventive measures to halt or mitigate the progression of radiation-induced brain injury.

## 5. AI in Histopathological Diagnosis

Histopathological examination has traditionally been considered the gold standard for diagnosing head and neck cancers. However, this method has its limitations. The diagnosis can be subjective, heavily reliant on the pathologist’s experience and expertise, and time-consuming due to the need for meticulous examination of tissue samples under the microscope. Furthermore, the increasing number of cancer cases globally has placed a significant burden on pathologists, potentially leading to delays in diagnosis. In this context, AI, particularly deep learning algorithms, has emerged as a powerful tool to augment and streamline histopathological diagnosis. AI’s capacity to analyze large datasets, recognize complex patterns, and provide consistent, objective results makes it a valuable asset in pathology.

### 5.1. Whole-Slide Image (WSI) Analysis

One of the key areas where AI has demonstrated significant promise is in the analysis of WSIs. WSIs are high-resolution digital images of tissue samples that allow pathologists to examine cells and tissue structures in great detail. However, manually reviewing these slides can be time-consuming, especially in large-scale cancer screenings. AI, particularly through CNNs, has shown remarkable capabilities in automating this analysis. Jeyaraj and Samuel Nadar, in 2019, obtained an accuracy of 91.4% (sensitivity 0.94; specificity 0.91) for 100 image datasets training for the classification of malignant tumors and normal tissue [16]. Additionally, the model analyzed slides at speeds far surpassing those of human reviewers, facilitating faster diagnostic workflows. These results underscore AI’s potential as a supplementary tool for pathologists, especially in high-volume clinical settings where speed and accuracy are crucial. Beyond detecting cancerous cells, AI models have also shown potential in identifying specific histopathological features, such as mitotic figures, necrosis, and differentiation patterns, which are important for tumor grading and treatment decisions. For example, recent studies showed that machine learning models may represent a helpful tool for the detection and grading of oral potentially malignant and malignant lesions [17,18,45].

### 5.2. Detection of Tumor-Infiltrating Lymphocytes (TILs)

TILs are critical biomarkers in cancer immunology. A high density of TILs in the tumor microenvironment is often associated with a better prognosis and a more favorable response to immunotherapy. In head and neck cancers, particularly those linked to HPV infection, the presence of TILs provides vital information about patient prognosis and treatment success. AI has been effectively employed to automatically quantify TILs in histological samples, which can significantly enhance the precision of immunotherapy decision-making. In a 2023 study by Wibawa et al., researchers developed an AI-based model to detect and quantify TILs through the analysis of WSIs of head and neck cancers, in particular nasopharyngeal carcinoma. This AI system assessed the spatial distribution and density of TILs with high precision, providing crucial prognostic information (for example, the risk of recurrence) that could guide personalized treatment strategies [19]. By automating TIL quantification, AI not only reduces the workload of pathologists but also minimizes subjective bias, leading to more consistent and reliable results.

### 5.3. AI in Identifying Rare or Subtle Histopathological Patterns

One of AI’s most impressive capabilities is its ability to detect rare or subtle histopathological patterns that may be overlooked by even the most experienced pathologists. For instance, AI models can be trained to identify early signs of tumor invasion or precursor lesions in head and neck cancers, which are often difficult to detect in routine histopathological exams. For examples, in a recent study by de Chauveron et al., an AI-based system detected early-stage OSCC in pre-malignant lesions with an accuracy greater than 90% [20]. Early detection is crucial, as timely intervention can significantly improve patient outcomes. Another area where AI excels is in detecting micrometastases—small clusters of cancer cells that have spread to lymph nodes but are often missed in traditional pathology. Recent studies found that AI models trained to analyze histological sections of lymph nodes in head and neck cancer patients may detect lymph node metastasis with an accuracy ranging from 78% to 91% [21]. Early detection of micrometastases can significantly influence treatment decisions, particularly in determining the need for adjuvant therapy.

### 5.4. AI-Assisted Tumor Segmentation and Quantification

Tumor segmentation, which involves delineating the boundaries of a tumor within a tissue sample, is a crucial step in cancer diagnosis and treatment planning. Accurate segmentation allows for the precise measurement of tumor size, depth of invasion, and involvement of surrounding tissues, all of which are essential for staging and determining the appropriate course of treatment. AI algorithms, particularly those based on CNNs, have shown great potential in automating this process, providing consistent and accurate results that aid in the staging of head and neck cancers [46].

### 5.5. Digital Pathology and AI Integration

With the growing adoption of digital pathology, integrating AI into the routine diagnostic workflow is becoming increasingly feasible. Digital pathology involves scanning and digitizing histological slides, making them accessible for analysis by AI systems. Several studies have demonstrated the effectiveness of AI-assisted digital pathology platforms in improving diagnostic accuracy, workflow efficiency, and reproducibility.

## 6. AI in Molecular and Genomic Analysis

In addition to imaging and histopathology, AI has been increasingly applied in the analysis of molecular and genomic data to enhance the diagnosis and prognosis of head and neck cancers. Molecular and genomic analyses are crucial for understanding the underlying biology of cancers, including those affecting the head and neck region. These analyses provide essential information about genetic mutations, molecular pathways, and tumor heterogeneity, which can influence prognosis and guide personalized treatment strategies. The vast volume and complexity of data generated from high-throughput genomic technologies, such as whole-exome sequencing, RNA sequencing, and proteomics, present challenges for traditional methods of analysis and interpretation. AI, particularly ML and DL algorithms, has emerged as a powerful tool for processing, analyzing, and integrating these large-scale molecular datasets. AI-driven molecular and genomic analysis holds the potential to improve the diagnosis, classification, and prognostication of head and neck cancers as well as to identify novel therapeutic targets.

### 6.1. Genomic Data Integration and Mutation Analysis

The integration of AI with genomic data is a rapidly advancing area in cancer research, particularly in the context of precision oncology. AI algorithms can process extensive datasets from sources such as TCGA to identify genetic alterations, molecular subtypes, and biomarkers associated with head and neck cancers. These models can reveal novel mutations or molecular signatures that conventional methods might miss. A recent study by Wang et al. developed a machine learning model to identify macrophage signatures, which may be helpful in predicting prognosis, immune infiltration, and immunotherapy response in head and neck cancer [22].

The model also uncovered new genetic markers not previously associated with disease aggressiveness, offering potential targets for future therapeutic interventions. Such AI-driven insights into the molecular mechanisms of head and neck cancer could lead to the development of targeted therapies tailored to individual genetic profiles. Another significant application of AI in genomic analysis is the identification of TMB, a biomarker used to predict responses to immunotherapy. High TMB is associated with a better response to immune checkpoint inhibitors.

### 6.2. Transcriptomic and Epigenomic Analysis

Beyond DNA-level alterations, RNA sequencing (transcriptomics) and epigenetic changes (such as DNA methylation and histone modifications) are critical in tumor development, progression, and treatment resistance. AI has been applied to these types of data to uncover molecular subtypes of head and neck cancers, predict patient outcomes, and identify potential therapeutic targets. A 2020 study by Serafini et al. analyzed RNA sequencing data from head and neck cancer patients, identifying different gene expression signatures, each with varying clinical outcomes and responses to therapy [23]. These findings illustrate AI’s potential to unravel the complex molecular heterogeneity of head and neck cancers and provide insights into patient stratification for personalized treatment. Additionally, AI has been used to analyze epigenetic modifications that regulate gene expression in cancer cells. Recent studies have analyzed AI’s performance in detecting HPV in head and neck cancer patients, distinguishing between HPV-positive and HPV-negative cancers with 79% sensitivity and 74% specificity [24]. This distinction is clinically significant, as HPV-positive head and neck cancers often have a better prognosis and respond differently to treatment compared to their HPV-negative counterparts. AI-driven epigenomic analysis thus holds promise for more precise classification of head and neck cancers, guiding treatment decisions and improving patient outcomes. However, at present, AI-driven epigenomic analysis to detect HPV needs to be improved, since its sensitivity and specificity do not match those of p16 immunohistochemistry [24].

### 6.3. AI in Liquid Biopsy Analysis

Liquid biopsies, which involve detecting ctDNA, CTCs, and other biomarkers in blood samples, have emerged as a non-invasive diagnostic tool with significant potential for early cancer detection, monitoring disease progression, and assessing treatment response. AI models have been developed to analyze the complex and often noisy data from liquid biopsies, improving the sensitivity and specificity of these assays. In a 2024 study, Hernando-Calvo et al. analyzed ctDNA profiles from liquid biopsies of patients with different types of cancer, including head and neck cancers [25]. This advancement represents a significant leap in cancer diagnostics, as traditional tissue biopsies are invasive and often challenging to obtain, particularly in recurrent or metastatic cases. Liquid biopsies combined with AI analysis offer a less invasive, more accessible approach to the early detection and monitoring of head and neck cancers, potentially improving survival rates through earlier intervention. AI-based analysis of liquid biopsy data has also shown promise in tracking MRD and predicting recurrence. A 2022 study by Flach et al. analyzed ctDNA levels in head and neck cancer patients post surgery and radiation therapy. The AI model accurately detected minimal residual disease, identifying patients at risk of recurrence before it became clinically apparent [26]. In their study, Hernando-Calvo et al. also found a correlation between gene expression in ctDNA and response to immunotherapy [25]. This application of AI in liquid biopsies could revolutionize post-treatment monitoring, enabling earlier interventions to prevent or address recurrences.

### 6.4. Multi-Omics Data Integration

A promising direction in applying AI to cancer genomics involves integrating multiple types of molecular data, often referred to as “multi-omics” analysis. This approach combines data from genomics (DNA mutations), transcriptomics (gene expression), proteomics (protein expression), and metabolomics (metabolic profiles) to provide a comprehensive understanding of tumor biology. AI models, particularly deep learning networks, are adept at handling the complexity of multi-omics data and extracting meaningful patterns that could guide personalized treatment strategies. In 2021, Wang and Li pointed out how using deep-learning-based AI models to integrate multi-omics data (including DNA mutations, RNA expression, and proteomic profiles) may represent a helpful tool for the early detection, outcome prediction, and identification of novel therapeutic targets in patients affected by head and neck cancer [27]. By integrating various layers of molecular data, AI has the potential to provide a more holistic view of tumor biology and enhance the precision of cancer treatment. Furthermore, integrating AI with multi-omics data is expected to improve the identification of biomarkers for cancer immunotherapy. A 2021 study by Feng and Hess analyzed how integrated multi-omics data may identify head and neck cancer patients likely to immune checkpoint inhibitors [28]. This approach could lead to more effective patient selection for immunotherapy, thereby reducing unnecessary treatments and improving overall outcomes.

## 7. Predictive Models for Patient Outcomes and Treatment Planning

The prognosis for HNCs can vary significantly due to factors such as the stage and location of the tumor, molecular characteristics, and patient comorbidities. Traditional prognostic methods often rely on clinical staging systems like the TNM classification, which, although useful, offer a limited perspective on tumor biology. These systems frequently overlook the intricate interactions among clinical, molecular, and environmental factors. In this context, AI has become a revolutionary tool for creating predictive models that integrate various types of data, providing a more accurate prediction of patient outcomes and aiding in treatment planning. AI-driven models can help clinicians identify patients at higher risk of recurrence or adverse outcomes and determine the most effective treatment strategies based on personalized risk profiles.

**Figure 2:**
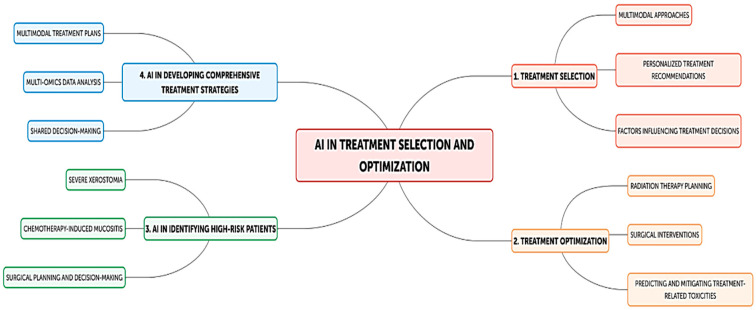
Flow diagram of potential AI usages in patient management. Abbreviations: AI, artificial intelligence.

AI has been employed in multiple areas, such as the following:

1. Prognosis and Risk Stratification: AI models predict survival, recurrence risk, and treatment responses by analyzing clinical, genomic, and histopathological data. For example, Mäkitie et al. developed a model to predict overall and disease-free survival in HNSCC [6], while Sultan et al. created a model for forecasting locoregional recurrence [29].
2. Response to Therapy: AI helps predict individual responses to treatments, such as surgery, radiation, chemotherapy, and immunotherapy. Models from Bang et al. predicted radiation therapy responses in head and neck cancer patients, with an accuracy reaching 87.5% [30], while Wang et al. integrated genomic and clinical data to forecast immunotherapy outcomes, identifying patients who were likely to benefit from immunotherapy [22].
3. Treatment Selection and Optimization: AI supports clinicians in selecting the best treatment combinations (Figure 2). Ahervo et al. demonstrated an AI-based decision-support system that optimized treatment planning (in particular radiotherapy) for advanced head and neck cancer patients [31].
4. Predicting Complications and Toxicity: AI models predict treatment-related complications, such as radiation-induced xerostomia or chemotherapy-induced mucositis, enabling adjustments to minimize side effects and improve patient care [32,33].

## 8. Comparative Analysis between AI Techniques in Head and Neck Cancer

ML, DL, and CNNs each offer unique strengths and challenges in terms of accuracy, practicality, and clinical relevance in HNC. ML works well with structured data, such as clinical records and genomic profiles, but it struggles with complex, unstructured data like medical images [6,47]. DL, on the other hand, excels in handling complex data types like imaging and histopathological slides, offering higher accuracy due to its ability to learn patterns automatically [6,47]. CNNs are particularly effective in image-related tasks like tumor detection and recurrence prediction, providing the highest accuracy in these areas [6]. In terms of practicality, ML is easier to implement, requiring less computational power and data, making it suitable for smaller clinics [6,47]. However, it sacrifices some accuracy compared to DL, which requires more resources and large datasets but can handle diverse data types simultaneously. CNNs are highly effective in medical imaging tasks but demand specialized hardware, making them less practical in resource-limited settings [6,47]. Regarding clinical relevance, ML models are commonly used in risk stratification and prognosis, with the added advantage of being more interpretable than DL models [6,47]. DL models, particularly CNNs, play a critical role in personalized treatment planning and medical imaging, but the complexity and “black box” nature of these models can limit their clinical adoption due to a lack of transparency.

ML offers ease of use and interpretability but falls short with complex data. DL models, especially CNNs, provide greater accuracy and clinical relevance in imaging but require more resources and are harder to interpret. The challenge lies in balancing practicality, accuracy, and trust in AI-driven insights for HNC care.

## 9. Challenges, Future Directions, and Conclusions

Different types of AI algorithms were tested in the included studies. While deep learning is more advanced than simple ML algorithms, this AI methodology introduces new challenges. DL requires larger datasets for training to develop an accurately performing model; so, if training datasets are limited or of poor quality (e.g., lack of heterogeneity), this may hinder the performance of deep learning [48].

The implementation of AI in diagnosing HNC presents significant technical, regulatory, and clinical challenges. Technically, AI models, particularly DL, require large, well-annotated datasets, which are difficult to standardize across institutions. Additionally, DL models often function as “black boxes,” making their decision-making process hard to interpret, a concern for clinicians who need transparency to trust AI-driven diagnoses. The computational demands of AI, especially for image-heavy tasks using CNNs, also limit their practicality in smaller healthcare settings. Regulatory challenges revolve around data privacy, security, and compliance with standards. Certifying AI models for clinical use is complex, particularly for evolving systems, and liability issues remain unresolved if AI diagnoses lead to errors. Clinically, trust in AI is a major barrier. Clinicians may hesitate to rely on AI tools without a clear understanding of their reasoning. AI models must also integrate seamlessly into existing clinical workflows without disrupting routine operations. Ensuring that these systems are generalizable and perform well across diverse patient populations is critical to their widespread clinical adoption.

Regarding future research needs, it is crucial to conduct longitudinal studies that assess the long-term impact of AI on patient outcomes. Understanding how AI-assisted diagnoses influence treatment decisions, disease progression, and survival rates is essential. Furthermore, research should prioritize the inclusion of diverse patient populations to ensure that AI models are trained on representative datasets, thus mitigating biases and improving the generalizability of solutions. Exploring the integration of AI with multidisciplinary approaches is also vital. Investigating how AI can enhance collaboration among oncologists, radiologists, pathologists, surgeons, and other specialists will contribute to the development of comprehensive and integrated care strategies.

Lastly, we cannot overlook the ethical and social implications associated with the adoption of AI in healthcare. It is essential to conduct research that addresses patient privacy concerns and the potential for algorithmic bias as well as to examine how the adoption of AI may affect equity and access to care for different populations.

In conclusion, while AI offers tremendous promise for advancing the diagnosis of HNC, a concerted effort is needed to address the current gaps in data standardization, model interpretability, and regulatory frameworks. By focusing on these areas and prioritizing future research that considers population diversity and ethical implications, we can enhance the integration of AI into clinical practice, ultimately improving early detection, treatment optimization, and patient outcomes in HNC care.

## Figures and Tables

**Table 1:** Summary of key original studies reporting AI integration in HNC.

Study	Advantages	Limitations
**Radiomics**		
Bologna et al. (2022) [11]	Relevance of ADC-based radiomics for prediction of response to induction chemotherapy in sinonasal cancer	Low number of cases compared with other studies
Wang et al. (2018) [12]	Pre-treatment MRI radiomics signatures can predict early response to induction chemotherapy in NPC	For patients with keratinizing squamous carcinoma and stage II patients, radiomic signatures cannot differentiate between responders and non-responders (due to the small number of these patients)
Zhao et al. (2020) [13]	Multiparametric MRI-based radiomics could be helpful for personalized risk stratification and treatment in NPC patients receiving induction chemotherapy	Relatively small number of patients; single-institutional nature of the study; as pathological examination of the treatment response was not possible in NPC patients, response evaluation only based on MRI might be less accurate
Romeo et al. (2020) [14]	ML analysis applied to CT-derived texture analysis features extracted from primary tumor was useful in predicting tumor grade and nodal status in patients with OSCC	Small sample size, which did not allow for further stratification of the N status and, particularly, for identifying N2c patients
Zhang et al. (2020) [15]	Three developed radiomic models can dynamically predict radiation-induced temporal lobe injury in NPC	Small number of radiation-induced temporal lobe injury-negative patients; use of 3D-CRT instead of IMRT; single-institution study
**Histopathology**		
Jeyaraj et al. (2019) [16]	Developed a DL algorithm based on partitioned CNN of automatic cancer diagnostic system to distinguish benign and malignant oral tumors	Not reported
Schmidl et al. (2024) [17]	First study using ChatGPT 4o ChatGPT 4.0 in the setting of recurrent/metastatic HNSCC	Lack of transparency of the resources; HNSCC cases of only one central European institution were investigated
Mahmood et al. (2020) [18]	Application and diagnostic accuracy of ML methods for detection and grading of pre-cancerous and cancerous head and neck lesions using WSI	Not reported
Wibawa et al. (2023) [19]	DL algorithm to detect tumor nuclei and lymphocyte nuclei in WSI to quantify TILs in NPC	Samples derived from a single cohort; the absence of information on lifestyle and comorbidities
De Chauveron et al. (2024) [20]	Highlighting the potential of DL, ML, and CNN for oral cancer detection using computer vision	Limited dataset; potential for false positives and false negatives; implementing other strategies when the model is uncertain about the pathology can enhance the accuracy and reliability
Adeoye et al. (2021) [21]	ML algorithms have a satisfactory accuracy for predicting nodal metastasis and prognosis of oral cavity cancer and malignant transformation of pre-malignant lesions	These models may not be streamlined enough for clinical application currently
**Molecular Analysis**		
Wang et al. (2024) [22]	Employing weighted gene co-expression network analysis to identify macrophage-related genes in HNSCC patients, which may predict prognosis, immune infiltration, and immunotherapy	Further verification needed from multiple centers; retrospective samples; lack of detailed information regarding subsequent therapy and follow-up
Serafini et al. (2020) [23]	Integration of multiple datasets through meta-analysis and growing integration among *omics* data	Low number of signatures associated with DNA methylation and with non-coding RNA
Song et al. (2023) [24]	Acceptable and promising performance of AI algorithms in predicting HPV status in HNC	AI algorithms not comparable to the routine p16 immunohistochemistry
Wang et al. (2021) [27]	Reviewing multi-omics image analysis of HNC using CNN and other DL neural networks; evaluating their application in early tumor detection, classification, prognosis, and metastasis prediction	Limited availability of well-characterized and adequately stored clinical tumor and non-tumor samples is a major challenge in proteomics and genomics studies; for multimodal learning, collecting data from the required modalities simultaneously could be problematic
Feng et al. (2021) [28]	Elucidating immune-related mutational landscapes and gene expression signatures by integrative analysis of multi-omics data and highlighting the potential therapeutic impact for HNC	Use of publicly available datasets from treatment-naive cancers; need for further studies
**Liquid Biopsy**		
Hernando-Calvo et al. (2024) [25]	Illustrating the potential of ctDNA as a biomarker of minimal residual disease detection and monitoring of early molecular-level recurrence in patients with HNSCC; feasibility of personalized ctDNA assays for therapy planning and follow-up	Unplanned retrospective analysis using tumor samples and ctDNA data from a single-institution, multi-histology clinical trial; small cohort; the time point to evaluate ctDNA dynamics before cycle 3 of Pembrolizumab based on results from previous studies
Flach et al. (2022) [26]	Illustrating the potential of ctDNA as a biomarker for detecting minimal residual disease and recurrence in HNSCC and demonstrating the possibility of using personalized ctDNA assays for detecting disease prior to clinical recurrence	Relatively small number of patients; ctDNA detection reported at the sample level, giving high sensitivity, but not informing on the levels and tracking of individual variants or the emergence of subclones
**Outcomes**		
Sultan et al. (2020) [29]	ML and DL methods designed to enhance prognostication of oral cancer, focusing on patient survival and locoregional recurrences	Further research at WSI level is needed
Bang et al. (2023) [30]	Use of AI models to predict therapeutic outcome and toxicity in the field of HNC radiotherapy	Non-systematic review method could cause potential risks of different biases with less transparency; study did not include a recently developed radiomics quality score
Ahervo et al. (2023) [31]	Use of AI to reduce treatment planning time and improve quality of radiotherapy allowing for dose reduction to the organs at risk	Potential selection bias
Iancu et al. (2021) [32]	Use of radiomic algorithms to predict the response to radiochemotherapy or induction chemotherapy and predict the risk of severe toxicities, especially xerostomia	Not reported
Rachi et al. (2023) [33]	Developing a program which can predict the occurrence of mucositis by reading DICOM data	Non-randomized trial; did not consider changes in BMI and dose as treatment progressed

Abbreviations: AI: artificial intelligence; HNCs: head and neck cancers; ML: machine learning; DL: deep learning; CNNs: convolutional neural networks; MRI: magnetic resonance imaging; WSI: whole-slide image; TILs: tumor-infiltrating lymphocytes; NLP: natural language processing; NPC: nasopharyngeal carcinoma; LASSO method: Least Absolute Shrinkage and Selection Operator; HNSCC: head and neck squamous cell carcinoma; SCC: squamous cell carcinoma; RF: Random Forest method; HPV: human papillomavirus; OSCC: oral squamous cell carcinoma; TCGA: The Cancer Genome Atlas; TMB: tumor mutational burden; ctDNA: circulating tumor DNA; CTCs: circulating tumor cells; MRD: minimal residual disease ADC: apparent diffusion coefficient; CRT: conventional radiation therapy; IMRT: intensity-modulated radiotherapy.

## Data Availability

No new data have been generated in the present article.

## Abbreviations

AI: artificial intelligence; HNC: head and neck cancers; ML: machine learning; DL: deep learning; CNNs: convolutional neural networks; MRI: magnetic resonance imaging; WSI: whole-slide image; TILs: tumor-infiltrating lymphocytes; NLP: natural language processing; NPC: nasopharyngeal carcinoma; LASSO method: Least Absolute Shrinkage and Selection Operator; HNSCC: head and neck squamous cell carcinoma; SCC: squamous cell carcinoma; RF: Random Forest method; HPV: human papillomavirus; OSCC: oral squamous cell carcinoma; TCGA: The Cancer Genome Atlas; TMB: tumor mutational burden; ctDNA: circulating tumor DNA; CTCs: circulating tumor cells; MRD: minimal residual disease ADC: apparent diffusion coefficient; CRT: conventional radiation therapy; IMRT: intensity-modulated radiotherapy.
